# Wild Edible Plants: A Challenge for Future Diet and Health

**DOI:** 10.3390/plants11030344

**Published:** 2022-01-27

**Authors:** Riccardo Motti

**Affiliations:** Department of Agricultural Sciences, University of Naples Federico II, Via Universita 100, 80055 Portici, NA, Italy; motti@unina.it

Wild edible plants (WEPs) can be defined as native species that grow and reproduce naturally in their natural habitat without being cultivated. Humans have gathered WEPs since ancient times, and they have become part of the human diet and traditional food systems. WEPs still play an important role when food crops are scarce, ensuring food sovereignty and food security, and they potentially contribute to well-being in vulnerable households. WEPs can also be central to efforts to empower local market actors and reduce the distance between consumers and producers, thereby diminishing the overreliance on globalized value chains [1]. Although the current global food system is capable of providing enough food for mankind, many still experience hunger or do not have access to a nutritious diet. On the other hand, the increased consumption of highly processed foods can negatively affect human health. Malnutrition (including over and undernutrition) is considered to be, in addition to climate change, a global threat, indicating an urgent need for a healthier and more sustainable food system. WEPs can therefore play an important role as an essential component of people’s diets in some regions of the world and provide greater dietary diversity for those who rely on them. In some cases, food plants are also eaten for their health-giving properties, and many species are commonly used as herbal medicines in folk phytotherapy for the treatment of several ailments [2]. Due to their clearly positive influence on health, they are often identified as functional foods, thanks to their higher contents of vitamins, phenols, flavonoids, antioxidants, microelements, and fiber than in cultivated crops. Wild plants are also perceived as a healthy alternative to cultivated vegetables that might be rich in pesticides and other chemicals. Therefore, wild species may have great potential as sources of unusual colors and flavors, bioactive compounds, and of dietary supplements. In recent decades, considerable evidence has emerged to support the hypothesis that diet and dietary factors play a major role in the occurrence of diseases. Recently, many studies have documented that the Mediterranean Diet (MD) meets several important criteria for a healthy diet. Since 2013, the MD has been recognized as Intangible Cultural Heritage of Humanity and, in recent years, has become a worldwide symbol for healthy food and lifestyle. The MD is high in nutrients, rich in taste, and heart healthy. By using healthy fats such as olive oil and improving endothelial function and blood pressure, the MD exerts a protective effect against the development of other chronic diseases such as diabetes and cancer. The MD includes wild plants in addition to cultivated fresh fruit and vegetables. Over the centuries, the former has constituted the main food ingredients in rural communities [3] and still represents a crucial, yet largely unknown, section of this diet. Finally, wild plants are an integral part of local cultural heritage and are often also consumed for their socio-economic and environmental sustainability. In other words, their consumption and gathering can provide cultural ecosystem services.

In this scenario, knowing processing techniques and the nutritional composition of wild food plants is as important as making an inventory of species. In recent decades, studies concerning WEPs used among rural populations have received growing attention, and many researchers have sought to analyze the persistence of traditional uses of plants and their products. Many floristic inventories regarding the use of WEPs at the local scale have been produced in Europe, the Americas, Africa, and Asia. In this context, the database for Italian WEPs is worthy of interest [4], in which 1103 taxa are documented to be used as alimurgic species. This paper provides a significant contribution to the knowledge of the wealth of uses of edible vascular plants in the whole of Italy, especially in light of their potential for cultural enhancement. Food plants in rural home gardens were also examined [5]; such knowledge is less extensively investigated in Europe, while it receives more attention in tropical areas. Some studies have focused on divergences in ethnobotanical and ecological knowledge in areas characterized by different linguistic communities or by past political borders, geographical, and cultural drivers [6].

These inventories play a fundamental role not only in knowledge concerning the local uses of plants but also in agrobiodiversity conservation and land protection strategies. The increased knowledge of WEPs could also have a useful impact on the agriculture of marginal areas, where it might be necessary to increase the availability of crops tolerant to extreme environmental conditions (high temperatures, low rainfall, or salinity) [7], taking into account the possible altered conditions of agriculture due to climate change. It should be emphasized that, in light of the above, the promotion of the use of WEPs could play a key role in the 2030 Agenda for Sustainable Development.

From the perspective that wild food plants are increasingly considered a potential source of natural healthy products, it is fundamental to foster biochemical research aimed at documenting their nutritional properties and main bioactive products [8,9]. The phytochemical and nutritional profiles of the species in question can therefore constitute basic knowledge for food pairing with other ingredients to improve nutritional and/or sensory quality and to find innovative cooking methods, allowing key molecules responsible for functional properties to be enhanced.

In conclusion, wild plants represent a crucial section of the human diet. It is hoped that an increasing amount of scientific research will focus on plant diversity, traditional knowledge, and agricultural studies and will foster bio-conservation strategies and sustainable food production. Biochemical knowledge is of crucial importance to evaluate the health benefits and physiological effects of WEPs in order to develop clinical investigations concerning their mechanisms of action, safety, and efficacy.

## Data Availability

All the relevant data used for the paper are in the text.
